# Multicenter Retrospective Registry Study on BCG Use in Non-Muscle Invasive Bladder Cancer in Latin America: BLATAM (Bladder Cancer in Latin America) Group

**DOI:** 10.1590/S1677-5538.IBJU.2024.0615

**Published:** 2025-01-23

**Authors:** Gustavo M. Villoldo, Matias Ignacio Gonzalez, Alvaro Vidal Faune, Ricardo Castillejos Molina, Luis Fernando Meza Montoya, José Gadu Campos Salcedo, Gonzalo Vitagliano, Hamilton Zampolli, Alcedir Raiser Lima, Ruben Bengio, Juan J. Camean, Germán Ándres Alfieri, Guido J. P. Escalante, Ivan Edgar Bravo Castro, Hernando Rios Pita, Juan Escuder, Francisco Rodriguez Covarrubias, Maria Fernanda de Oliveira, Rafael Sanchez-Salas, Gabriel Andrés Favre, Eduardo Guevara, Esteban Arismendi Videla, Guillermo Martinez Delgado, Ignacio Tobia, Roberto F. Villalba Bachur, Ana Maria Autran

**Affiliations:** 1 Instituto Alexander Fleming Buenos Aires Argentina Instituto Alexander Fleming, Buenos Aires, Argentina; 2 Hospital Italiano Buenos Aires Argentina Hospital Italiano, Buenos Aires, Argentina; 3 Fundación Arturo Lopez Perez Santiago Chile Fundación Arturo Lopez Perez, Santiago, Chile; 4 Instituto Nacional de Ciencias Médicas y Nutrición Salvador Zubirán México Instituto Nacional de Ciencias Médicas y Nutrición Salvador Zubirán, México; 5 Clínica Oncosalud Lima Perú Clínica Oncosalud, Lima, Perú; 6 Hospital Central Militar México Hospital Central Militar, México; 7 Hospital Alemán Buenos Aires Argentina Hospital Alemán, Buenos Aires, Argentina; 8 Instituto de Câncer Dr. Arnaldo Vieira de Carvalho São Paulo Brasil Instituto de Câncer Dr. Arnaldo Vieira de Carvalho, São Paulo, Brasil; 9 Hospital Regional do Vale do Paraíba Taubaté SP Brasil Hospital Regional do Vale do Paraíba, Taubaté, SP, Brasil; 10 McGill University Health Center Canada McGill University Health Center, Canada; 11 Centro Urológico profesor Bengió Córdoba Argentina Centro Urológico profesor Bengió, Córdoba, Argentina; 12 Universidad Veracruzana Instituto de Urologia Latinoamericano Mexico Instituto de Urologia Latinoamericano (UROLATAM), Universidad Veracruzana, Confederación Americana de Urologia

**Keywords:** Urinary Bladder Neoplasms, Non-Muscle Invasive Bladder Neoplasms, BCG Vaccine

## Abstract

**Objectives:**

This study, conducted by the Bladder Cancer in Latin America (BLATAM) group, aims to analyze epidemiological and therapeutic data on non-muscle invasive bladder cancer (NMIBC) in Latin American patients. It seeks to identify factors contributing to suboptimal responses to Bacillus Calmette-Guérin (BCG) therapy and assess areas for improvement in regional treatment practices.

**Materials and Methods:**

A multicenter retrospective study was carried out in collaboration with reference Urology Departments across Latin America. Data were collected using an electronic Case Report Form (CRF) from 2011 to 2021, capturing demographics, clinical presentation, treatment details, and follow-up of NMIBC patients treated with BCG. Statistical analyses included Kaplan-Meier survival analysis for relapse-free survival (RFS).

**Results:**

Data from 292 patients across five countries were analyzed, with a mean age of 70.3 years and a male prevalence of 74%. Smoking history was reported in 70.6% of patients. The mean time to the first BCG dose was 2.4 months post-TURBT, with 26.7% of patients exceeding the recommended 60-day window for induction initiation. While 84% of patients completed BCG induction, only 45.9% followed the recommended Lamm maintenance schedule. Delays in starting maintenance cycles were observed, with a median delay of over 36 days for the first cycle and 65 days for the second cycle. RFS at 1 year and 5 years for high-risk patients was 87.3% and 53.3%, respectively.

**Conclusions:**

This study highlights critical deviations from recommended NMIBC management protocols in Latin America, including delayed BCG initiation and inconsistencies in maintenance therapy. These findings emphasize the need for standardized treatment protocols and improved adherence to international guidelines, which could enhance NMIBC patient outcomes in the region. Collaborative efforts are essential to develop region-specific strategies, improve data collection, and ultimately provide better care for bladder cancer patients in Latin America.

## INTRODUCTION

Bladder cancer ranks among the most prevalent malignancies worldwide, with a notably increasing incidence in recent years (1–3). The disease's multifaceted clinical presentation and diverse therapeutic approaches pose significant challenges for healthcare systems and clinicians globally.

Within the spectrum of bladder cancer, non-muscle invasive bladder cancer (NMIBC) predominates as the most common subtype. Characterized by its non-invasive nature, NMIBC presents substantial management dilemmas, especially regarding its high recurrence rate and the progression to muscle-invasive disease (4).

Intravesical Bacillus Calmette-Guérin (BCG) immunotherapy has emerged as a cornerstone in the management of high-risk and intermediate-risk NMIBC, demonstrating remarkable efficacy in reducing recurrence and progression rates. However, a substantial proportion of patients fail to achieve a satisfactory response to BCG (5, 6), underscoring the need for tailored therapeutic approaches and predictive biomarkers.

In Latin America, the challenges posed by NMIBC (non-muscle-invasive bladder cancer) and BCG therapy are exacerbated by unique regional factors, such as disparities in healthcare access, genetic heterogeneity, and socioeconomic determinants (7–9). Despite these challenges, the marked paucity of complementary data hinders the analysis of regional differences associated with treatment efficacy.

Therefore, our hypothesis was that regional differences in Latin America, including disparities in healthcare access and genetic and socioeconomic particularities, are associated with a lower response to BCG therapy in patients with NMIBC.

This study, facilitated by the creation of the first collaborative group of Latin American urologists dedicated to bladder cancer (BLATAM), aims to collect relevant epidemiological and therapeutic data on the disease, as well as to identify factors that may contribute to a suboptimal response to BCG therapy in Latin American patients.

The information obtained from this study promises to reveal local therapeutic weaknesses, which could contribute to the implementation of regional health policies to improve outcomes for NMIBC patients in Latin America.

## MATERIALS AND METHODS

A multicenter retrospective cohort study was conducted by inviting members of reference Urology Departments from the Latin American Confederation of Urology (CAU) and members of the BLATAM group (Bladder Cancer in Latin America) to participate. Participating centers were asked to complete an electronic Case Report Form (CRF) provided by the CAU during the 12 months of 2021, which contained clinical, pathological, treatment, and follow-up data for patients with NMIBC treated with BCG in the 10 years prior to the study initiation in 2021. Patients with inconsistencies in treatment data were excluded. The protocol was reviewed by the CAU Ethics Office, protocol number 002/2020, no objections were found, and given that anonymous retrospective data would be entered, approval from local Ethics Committees was not required.

Through the electronic CRF, demographic data such as Country and Treatment Center, age, gender, smoking history, as well as information on the original bladder tumor (histological type, grade, previous treatments, number and size of tumors, and performance of re- transurethral resection of bladder tumor [TURBT]) were collected. Follow-up data included time to BCG induction, frequency and intervals of administration, type of BCG regimen, number of doses received, and occurrence of recurrence or progression.

To measure time to the start of induction (six weekly instillations for six weeks), the interval from TURBT to the first dose of BCG was considered, and in cases of re-TURBT, the time from re-TURBT to the start of the first dose of BCG was taken. For time to recurrence or progression, the interval from the first dose of BCG to the diagnosis of the event or the last visit was considered. Recurrence was defined as the appearance of a new tumor diagnosed during follow-up by cystoscopy, while progression referred to the appearance of invasive tumor or distant disease.

For all cases, the frequency of BCG administration was requested. In addition, the frequency of oncological evaluations and the methods employed (cystoscopy, image, cytology, and others) were recorded.

The optimal time for maintenance regimens was considered to be according to the Lamm schedule: three weekly instillations for three weeks at 3, 6, 12, 18, 24, 30, and 36 months from the start of the first induction BCG ± 15 days. For example, the first maintenance cycle should be at 3 months or 90 days from the first induction BCG instillation, with a window for receiving this maintenance from day 75 to 105 (90 days ± 15 days).

## Statistical Analysis

Convenience sampling was used given the characteristics of the study. Continuous variables are presented as mean and standard deviation (SD) or median and interquartile range (IQR) according to the distribution. Categorical variables are summarized as absolute value and percentage (%). Kaplan-Meier survival analysis was used to estimate 1- and 5-year survival, along with a 95% confidence interval (95% CI). The software used was SPSS 22.0™ (IBM Corp, New York, USA).

## RESULTS

Of a total of 454 cases entered into the electronic Case Report Form (CRF), 292 were eligible for inclusion in the study. The percentages of included patients by country were as follows: Argentina (56.4%), Mexico (15.5%), Chile (14.4%), Peru (9.9%), and Brazil (3.8%). Four centers from Argentina (Instituto Alexander Fleming, Hospital Italiano de Buenos Aires, Hospital Alemán de Buenos Aires, and Centro Bengió), 2 from Mexico (Instituto Nacional de Ciencias Médicas y Nutrición "Salvador Zubirán" and Hospital Central Militar), 2 from Brazil (Instituto Armando Viera de Carvalho and Hospital Regional Paraiba), 1 from Chile (Fundación Arturo López Pérez), and 1 from Peru (Clínica Oncosalud AUNA) participated.

The age range of included population was 20 to 98 years (mean 70.3, SD 11.2), with 216 patients (74%) being male and 76 (26%) females. Regarding medical history, 206 cases (70.6%) were current or former smokers, and 10 (3.3%) had exposure to toxins such as anilines or similar substances. Regarding the clinical presentation of bladder cancer, hematuria was the most common, with 219 cases (75%) (Table-1).

**Table 1:** Descriptive anlaysis: Diagnosis, TNM, Histology, tumor size, Transurethral resection of bladder tumor (TURBT) quality and BCG treatment characteristics.

Diagnosis (%)		Total (n 292)
	Haematuria	219 (75)
	Incidental	35 (12)
	Urinary tract infection	2 (0.7)
	Others	36 (12.7)
**Histology (%)**		
	Urotelial	287 (98.2)
	Urotelial, glandular differentiation	3 (1)
	Micropapilar	2 (0.8)
**High grade (%)**		215 (73.6)
**T Stage (%)**		
	pTa	84 (28.8)
	pT1	171 (58.6)
	CIS	7 (2.4)
	pT1+CIS	16 (5.5)
	Others	14 (10.3)
**Tumor Size**		
	Multiple tumors (%)	102 (34.9)
	Largest tumor < 3 cm (%)	190 (65.1)
	High risk (%)	193 (66.1)
**TURBT quality (%)**		
	Muscle in TURBT	232 (79.5)
	Re TURBT	167 (57.2)
**BCG Schedule**		
	Started Induction	268 (91.8)
	Finished Induction	245 (84)
	No Maintenance	59 (20.2)
	LAMM Maintenance	134 (45.9)
	No LAMM Maintenance	99(33.9)
**BCG doses**		
	0-1 doses	120 (41)
	2-9 doses	126 (43.1)
	>10 doses	46 (15.8)
**BCG Intolerance**		26 (8.9)

In 201 cases (68.8%), the diagnosis was primary (non-recurrent) cancer. The most common form of presentation at the time of initial TURBT was lesions smaller than 3 cm in 65.1% of cases.

The vast majority of patients had histopathological diagnosis of urothelial carcinoma (98.2%), with a minimal number of reports describing histological variants (urothelial with glandular differentiation and micropapilar). Post-initial TURBT staging revealed 28.8% of patients with pTa disease, 58.6% with pT1, a small proportion with CIS of 2.4%, and the combination of pT1/CIS in 5.5%. A total of 73.6% of patients presented with high-grade disease, with 66.1% classified as high-risk according to the 2020 EAU guidelines. The percentage of patients who underwent re-TURBT was 57.2%, as shown in Table-1.

Regarding the quality of the initial TURBT, detrusor muscle was reported in the specimen of 232 patients (79.4%). A re-TURBT was performed in 167 patients, corresponding to 57.2%.

Among patients indicated for intravesical BCG treatment, 268 (91.8%) started induction with a weekly instillation for 6 weeks, only 245 patients (84%) completed it; therefore, only 7.8% of patients did not complete the full regimen of six instillations.

Of the total number of patients, only 45.9% received BCG according to LAMM Maintenance schedule, 33.9% followed a different maintenance regimen, and 20.2% did not receive any maintenance treatment.

The median time to initiation of induction was 1 month (IQR 0-2) (mean time 2.4 months), with 78 cases (26.7%) where the initiation of induction exceeded 60 days. The first maintenance cycle was initiated in 154 patients. The median time to the first maintenance cycle was 126 days (IQR 100-161). Notably, 89.4% of patients began the first maintenance cycle outside the 90-day ± 15-day window, with an average delay of more than 36 days. Among the 118 patients with a reported second maintenance regimen, the median time to initiation was 245 days (IQR 203-294), where 93.2% started outside the 180-day ± 15-day window, with an average delay of 65 days. During the follow up, the median number of doses administered post induction was 3 (IQR 0-9) with only 74 patients (25.3%) receiving 9 or more doses. A total of 29 patients (8.9%) experienced BCG intolerance resulting in treatment interruption (Table-1).

The mean follow-up time was 22.5 months (IQR 12-50), with significant patient loss during this period, as 28.8% of patients did not reach the one-year follow-up. During this time, 69 recurrences (23.6%) were documented. Of the patients who were followed for at least one year, 65% did not receive 9 post-induction doses of BCG.

**Figure 1:**
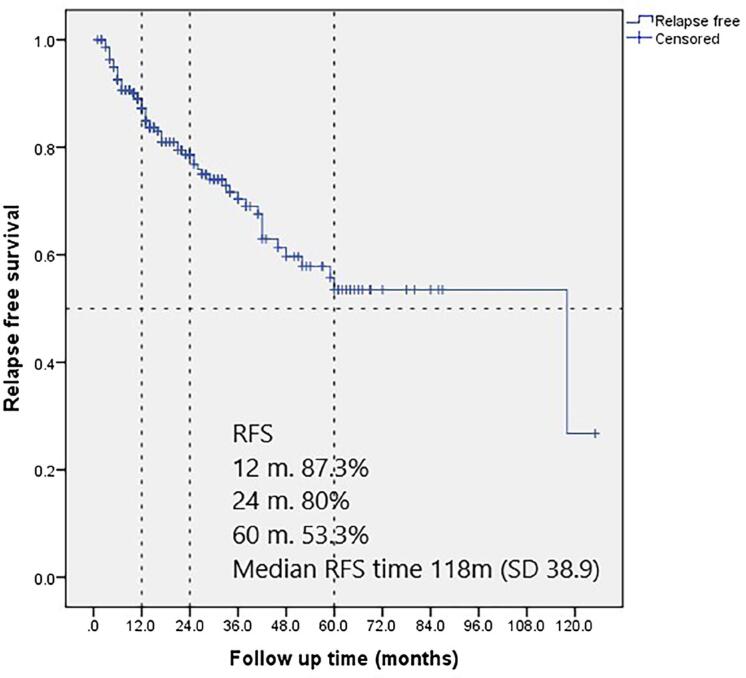
Kaplan Meier curve. Relapse free survival (RFS) and medial survival time in high-risk patients.

Among the 222 high-risk patients assessed, the 1-year relapse-free survival (RFS) rate was 87.3% (95% CI 82.8-91.8), the 2-year RFS rate was nearly 80% and the 5-year RFS was 53.3% (95% CI 42.4-64.6) (Figure-1).

Reported treatment for recurrence cases included: additional BCG regimens in 22 cases (31.9%), mitomycin in 5 cases (7.2%), and gemcitabine, or inclusion in clinical trials in 4 cases (5.8%). Cystectomy was performed in nine cases (13%), while treatment was not reported in 28 cases.

Regarding follow up cystoscopies, 85% of patients (248) underwent the first cystoscopy at 3 months, with a decreasing number at 6 months (175 patients, 60%), 9 months (147 patients, 50%), and only 100 (34%) at 12 months.

Urine cytology was taken as follows: 140 PAPs at 3 months (56.5%), 112 (38%) at 6 months, 81 (28%) at 6 months, and 71 (24%) at 12 months.

## DISCUSSION

Bladder cancer has distinct characteristics that differentiate it from other types of cancer, primarily due to the difficulty in predicting its behavior. Not only does it have a high recurrence rate, but in some cases, it can potentially progress to more advanced stages. Bladder Carcinoma In Situ (CIS), unlike other locations, can behave aggressively, with a high probability of progressing to the deeper layers of the bladder wall if not treated adequately (10).

Our study provides valuable information on the clinical presentation, epidemiological aspects of non-muscle-invasive bladder cancer (NMIBC) in Latin American patients, and how these patients are treated in this region. While our findings, for the most part, agree with established global trends regarding the prevalence of initial diagnostic symptoms and the widespread use of Bacillus Calmette-Guérin (BCG) therapy, our analysis reveals inconsistencies related to the quality of treatments.

Our data of age and gender are consistent with the global trend of a higher incidence in men. However, it is interesting to note that, globally, incidence rates increase more sharply between the ages of 50 and 54, especially in men (11).

The use of tobacco is the most frequent risk factor and is associated with approximately 50% of bladder cancer cases (11, 12), in our registry, a higher percentage of 70.6% was found to be related to smoking.

Our findings agree with those reported by Ramírez et al. (13), who found that gross hematuria was the most common presenting symptom in 75% of our bladder cancer patients, a figure similar to the 78.3% described in their study. This high prevalence of gross hematuria at diagnosis reinforces the importance of this clinical manifestation as a warning sign. Additionally, our data corroborate the association between gross hematuria and more advanced pathological stages, underscoring the need for early detection to improve prognosis.

Regarding reported histological variants, our detection rate was 1.8%, significantly lower than the average of 25% described in literature. The results obtained underscore the crucial importance of having specialized pathologists in multidisciplinary teams (14) to optimize diagnosis and, ultimately, patient outcomes. Similarly, our detection rate of carcinoma in situ (CIS) was 7.9%, lower than the reported (10%) (14, 15). These findings highlight the potential of advanced imaging technologies such as blue light cystoscopy, although its high cost limits its implementation in Latin America.

The quality of the first transurethral resection of the bladder (TURB) is fundamental to the management of bladder cancer. The presence of detrusor muscle in the specimen, which in our series was 80%, is a key indicator of an adequate resection and has been associated with better oncological outcomes, including recurrence time and cancer-specific survival (16, 17). However, the percentage of patients who required a re-TURB in our cohort (57%) suggests that there is still room for improvement. Recent studies, such as the NIMBUS trial (18), have demonstrated that a higher rate of re-TURB, close to 90%, can improve oncological outcomes. These findings highlight the importance of following current guidelines and considering re-TURB more aggressively, especially in cases of incomplete resections or pT1 lesions.

Our analysis revealed significant deviations from established treatment guidelines for NMIBC, particularly regarding the initiation of intravesical BCG therapy. In our study, the mean initiation of the first induction dose of BCG was 2.4 months post-TURBT, considerably later than recommended in pivotal studies such as SWOG 8507 (6) and EORTC 30962 (6,19), where initiation was recommended 15 days post-TURBT. This delay in treatment initiation could compromise the efficacy of BCG and oncological outcomes. It is essential to align our clinical practices with international recommendations to optimize the management of these patients.

Maintenance schedules with BCG have been shown to be essential in preventing bladder cancer recurrence. However, our results indicate that a significant percentage of patients did not receive the recommended number of BCG instillations according to established regimens, such as the Lamm protocol. 84.3% of our patients received less than 9 maintenance doses in the first year, and furthermore, significant delays in the initiation of maintenance cycles were observed. These deviations from clinical guidelines could compromise treatment efficacy and increase the risk of recurrence.

Despite protocols recommending 15 BCG instillations during the first year, as demonstrated in NIMBUS trial, our findings indicate suboptimal adherence to treatment. This lack of compliance with established guidelines raises concerns about the efficacy of intravesical therapy and could increase the risk of recurrence and disease progression. Delays in the initiation of treatment and deviations in dosing regimens may compromise the immune response induced by BCG and consequently affect oncological outcomes.

In our study, we detected a considerable patient lost to follow up. Despite this characteristic, the RFS results were not so distant from those reported. However, it is not intended to emphasize that by performing an incorrect scheme or follow-up, the same results are obtained as when performing the scheme correctly. Our study is biased due to all the factors described above, but mainly because of the limited recording of recurrences, the significant loss of follow-up, and the large amount of missing data we encountered. This RFS should not be considered as an indicator that good results can be achieved by doing things incorrectly. Instead, it should serve as a reminder to improve our treatments and report data more accurately.

A significant limitation of our study relies on the inability to accurately quantify the proportion of non-responders to BCG, reflecting the lack of standardized follow-up protocols in the region. This observation highlights the need for more robust monitoring systems to evaluate treatment response. In parallel, recent years have witnessed an evolution in the management guidelines for non-muscle-invasive bladder cancer, with a greater emphasis on the continuity of BCG treatment even in the face of low-grade recurrences. This new perspective, supported by growing evidence, seeks to optimize outcomes for patients. Additionally, the incorporation of office-based electrocoagulation has expanded therapeutic options for the management of low-grade lesions, improving patient accessibility and quality of life (20).

Treatment protocols for bladder cancer have been significantly impacted by two several factors. The COVID-19 pandemic, disrupting in healthcare and compromising treatment adherence and highlighting the need to develop more flexible and adaptable care systems. Secondly, the recurrent global shortage of the drug (19, 21), this scarcity can be attributed to various factors, including increased demand, production constraints, and regulatory hurdles (20, 22). The lack of access to BCG is heterogeneous across the region, being more evident in some countries, such as Brazil (8).

The adoption of electronic health records (EHRs) in Latin American healthcare institutions lacks a homogeneous planning. While it has improved operational and registration efficiency, it has introduced new challenges for research. Data fragmentation, lack of compatibility between systems, and the loss of historical information during the transition have limited the ability to perform comprehensive retrospective analyses. These inherent limitations of EHRs have affected the robustness of our findings, demonstrating the need to develop standards for transitions and the analysis of clinical data in the region.

The heterogeneity in the management of NMIBC in Latin America and the challenges identified in our study underscore the need to strengthen research in this area. Through collaborative research, we can develop more effective treatment protocols, identify predictive biomarkers of response, and evaluate the impact of interventions on patients’ quality of life. It is essential to invest in research to improve outcomes for NMIBC patients in our region.

Therapeutic options have traditionally been limited, primarily relying on intravesical Bacillus Calmette-Guérin (BCG) and mitomycin C (MMC). Currently, treatment options remain constrained, with the only approved systemic therapy being intravenous pembrolizumab and the sole approved intravesical therapy being nadofaragene firadenovec, both indicated for BCG-unresponsive carcinoma in situ (CIS) in the United States. However, research in this field is highly active, with numerous promising alternative therapies under development that have the potential for regulatory approval in the near future. For intermediate-risk NMIBC, a preference exists for ablative approaches over adjuvant therapies. In contrast, for high-risk NMIBC, clinical trials predominantly focus on investigating alternatives to BCG, such as substituting BCG entirely or minimizing patient exposure by employing induction-only courses, often in conjunction with novel therapeutic agents.

## CONCLUSIONS

In conclusion, the need to consolidate collaborative registries in Latin America is imperative. By joining efforts and collecting data more efficiently, we can obtain a more accurate and up-to-date view of the clinical reality in our region. This will not only enrich our caseload and pathological diagnoses but also evaluate the efficacy of implemented therapeutic alternatives. It is essential to promote the creation of new prospective studies with a larger number of patients and long-term follow-up to strengthen the scientific evidence and improve care for our patients.

## Data Availability

The information was obtained from patient's medical records
